# Adeno-Associated Virus-Based Gene Therapy for Lafora Disease in *Epm2b*-Deficient Mice

**DOI:** 10.3390/ijms262411930

**Published:** 2025-12-11

**Authors:** Luis Zafra-Puerta, Nerea Iglesias-Cabeza, Pascual Sanz, María Adelaida García-Gimeno, Gema Sánchez-Martín, Marina P. Sánchez, José M. Serratosa

**Affiliations:** 1 Laboratory of Neurology, Instituto de Investigación Sanitaria-Fundación Jiménez Díaz, Universidad Autónoma de Madrid (IIS-FJD, UAM), 28040 Madrid, Spain; 2 Fondazione Malattie Rare Mauro Baschirotto BIRD Onlus, 36023 Longare, VI, Italy; 3 Instituto de Biomedicina de Valencia, CSIC, Jaime Roig 11, 46010 Valencia, Spain; 4 Centro de Investigación Biomédica en Red de Enfermedades Raras (CIBERER), 46010 Valencia, Spain; 5 Department of Biotechnology, Escuela Técnica Superior de Ingeniería Agronómica y del Medio Natural, Universitat Politécnica de València, 46022 Valencia, Spain

**Keywords:** Lafora disease, progressive myoclonic epilepsy, *Epm2b*^−/−^ mouse, malin, gene therapy, rAAV

## Abstract

Lafora disease is a fatal neurodegenerative disorder caused by loss-of-function mutations in the *EPM2A* or *EPM2B* genes, which encode laforin and malin, respectively. These mutations lead to the accumulation of intracellular inclusions of abnormal glycogen, known as Lafora bodies, the hallmark of the disease. Symptoms typically begin in early adolescence with seizures and rapidly progress to cognitive and motor decline, ultimately resulting in dementia and death within a decade of onset. Disruption of *Epm2a* or *Epm2b* in mice causes neuronal degeneration and Lafora body accumulation in the brain and other tissues. *Epm2a*^−/−^ and *Epm2b*^−/−^ mice exhibit motor and memory impairments, epileptic activity, and molecular and histological abnormalities. We previously demonstrated that intracerebroventricular delivery of a recombinant adeno-associated virus carrying *EPM2A* significantly improved pathology in *Epm2a*^−/−^ mice. In this study, we tested recombinant adeno-associated virus-mediated delivery of the human *EPM2B* gene in *Epm2b*^−/−^ mice. The treatment partially improved neurological, molecular, and histopathological outcomes, although some pathological features persisted. Importantly, our findings reveal differences between *EPM2A*- and *EPM2B*-based gene therapies, highlighting the need to better understand their distinct mechanisms. Despite limitations, our study provides new insights into the complexity of targeting *EPM2B* mutations in Lafora disease.

## 1. Introduction

Lafora disease (LD) (OMIM#254780; ORPHA:501) is a rare, progressive neurodegenerative disorder that initially presents with cognitive deficits, myoclonus, and visual and generalized tonic–clonic seizures [1,2,3]. Onset typically occurs in late childhood or early adolescence, followed by a rapid decline leading to severe dementia, ataxia, dysarthria, aphasia, visual loss, and respiratory failure. This progressive deterioration ultimately results in a vegetative state and death, usually within a decade of symptom onset [4,5,6,7]. LD is caused by more than 100 recessive mutations in the *EPM2A* gene (OMIM: 607566) [8,9,10], which encodes laforin, a dual-specificity phosphatase [11], or in the *EPM2B*/*NHLRC1* gene (OMIM: 608072) [12,13], which encodes malin, an E3 ubiquitin ligase [14]. Malin contains a RING (Really Interesting New Gene) zinc finger domain, which confers E3-ubiquitin ligase activity, and six NHL domains (NCL1, HT2A, and LIN-41), enabling protein–protein interactions [14]. While malin promotes the degradation of laforin through the ubiquitin–proteasome system (UPS) [14,15], laforin stabilizes malin [16,17]. Recently, it has been shown that malin depends on laforin to interact with other proteins involved in glycogen metabolism [16]. Together, laforin and malin form a functional complex that regulates glycogen metabolism, maintains protein homeostasis, and supports mitochondrial function, oxidative stress regulation, and autophagy [15,16,18,19,20,21,22,23,24,25]. A deficiency in this complex leads to the formation of Lafora bodies (LBs), the hallmark of the disease. These LBs consist of polyglucosans—abnormal, insoluble, and poorly branched forms of glycogen—along with a protein content of 6% to 28% [20,26,27,28,29], placing LD within the family of glycogen storage diseases.

Mouse models lacking laforin (*Epm2a*^−/−^) [30] or malin (*Epm2b*^−/−^) [31] expression exhibit neurological alterations that closely resemble those observed in patients with LD, including the presence of LBs, dyskinesia, impaired motor activity and coordination, deficits in episodic memory, and epileptic activity. These models also display hyperexcitability, as evidenced by heightened response to the convulsant agent pentylenetetrazol (PTZ) [30,31,32,33]. They have proven valuable for testing potential treatments aimed at alleviating or curing LD symptoms. Recently, we reported that metformin, an AMPK activator [34], produced promising outcomes in these models [35,36,37]. As a result, metformin is now administered globally to patients, helping delay neurological symptoms and improve daily activities [36,38]. Additionally, alternative therapeutic strategies, including sodium selenate [39], VAL-0417 [40,41], antisense oligonucleotide (ASO) targeting Gys 1 [42,43], Myozyme^®^ [44], and other neuroinflammation modulators [45], have been evaluated in these animal models.

We recently demonstrated that a recombinant adeno-associated virus (rAAV) carrying the coding region of the human *EPM2A* gene (rAAV2/9-CAG-h*EPM2A*) significantly reduced neurological and histopathological abnormalities, diminished epileptic activity, and decreased the formation of LBs in the *Epm2a*^−/−^ mouse model [46]. Here, we applied gene therapy using a rAAV vector carrying the human *EPM2B* (h*EPM2B*) gene under the control of a CAG promoter, which drives general, widespread expression, in *Epm2b*^−/−^ mice through a single intracerebroventricular (ICV) injection. This treatment led to significant improvements in key neurological and histopathological parameters, although some pathological hallmarks of the disease were less responsive. Western blot analysis revealed beneficial changes in several molecular mediators associated with neuroinflammation and cell death, with these partial improvements persisting up to nine months after ICV injection. Notably, compared to our previously reported results with *EPM2A* gene therapy [46], the outcomes achieved through *EPM2B* restoration were less robust. These findings underscore critical differences between *EPM2A*- and *EPM2B*-based gene therapy approaches and highlight the need for further investigation to elucidate the mechanisms underlying this divergent therapeutic response.

## 2. Results

### 2.1. ICV Injections of the rAAV-hEPM2B or rAAV-GFP Vectors Enable Efficient Brain Transgene Transcription and Translation

**Figure 1:**
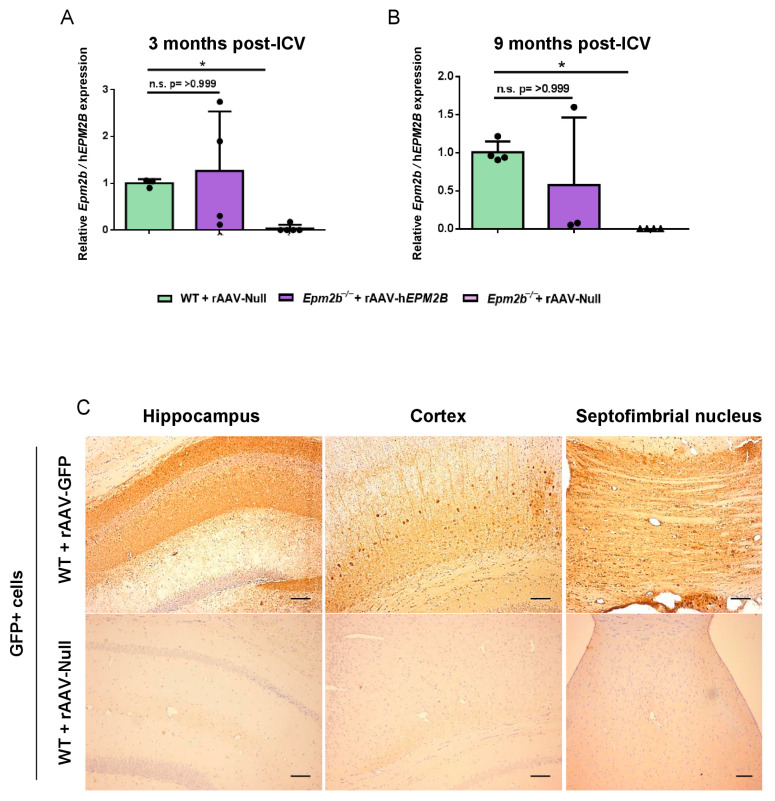
Analysis of h*EPM2B* transcription and GFP expression in the brain 3 and 9 months after a single ICV injection of the rAAV-h*EPM2B* or rAAV-*GFP* vectors: (**A**,**B**) RT-qPCR quantification of *Epm2b* cDNA levels in the brains of WT, and h*EPM2B* cDNA levels in the brains of *Epm2b*^−/−^ mice, 3 (**A**) and 9 (**B**) months post-injection, normalized to housekeeping *Gapdh* levels. Data are shown as mean ± SD. (**C**) Biodistribution of the rAAV2/9 vector in the brain of WT mice 3 months after ICV injection of the rAAV-*GFP* vector, analyzed by immunohistochemistry with a GFP antibody. Left panel, hippocampus; central panel, cortex; right panel, septofimbrial nucleus. * *p* < 0.05. *n* = 3–5 mice per group Scale bar = 100 µm.

Three and nine months after a single ICV injection of rAAV-h*EPM2B* or rAAV-Null vectors into the brains of 3-month-old *Epm2b*^−/−^ and WT mice, the expression of the h*EPM2B* transgene was measured using RT-qPCR (Figure 1A,B). The results showed that levels of h*EPM2B* transcripts in *Epm2b*^−/−^ mice, at both 3 and 9 months post-injection, were comparable to the levels of *Epm2b* transcripts in WT animals. Due to the lack of effective antibodies for detecting the malin protein, we used a rAAV2/9 vector with the CAG promoter carrying the *GFP* reporter gene to analyze vector biodistribution in the central nervous system. Three months after ICV injection of the rAAV-*GFP* vector in WT mice, immunohistochemical analysis revealed widespread GFP expression throughout the hippocampus, cortex, and the septofimbrial nucleus (Figure 1C). This indicates that a single ICV injection of the rAAV-h*EPM2B* or rAAV-*GFP* vectors enables the transcription of the h*EPM2B* or *GFP* transgenes and the translation of the GFP protein (and, by inference, the malin protein) within the central nervous system of *Epm2b*^−/−^ and WT mice.

### 2.2. The rAAV-hEPM2B Vector Does Not Reduce LB Formation in the Brains of Epm2b^−/−^ Mice

**Figure 2:**
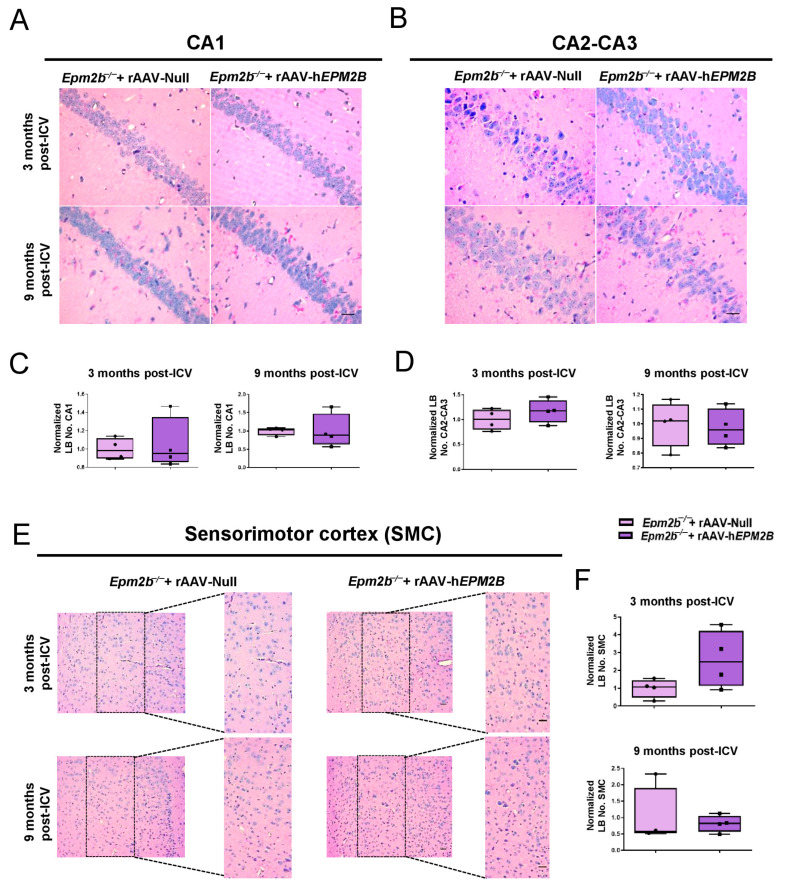
Quantification of LB formation in the brains of *Epm2b*^−/−^ mice treated with rAAV-h*EPM2B* or rAAV-Null: (**A**,**B**,**E**) PAS-D staining in the CA1 (**A**) and CA2-CA3 (**B**) regions of the hippocampus, and in layers IV-V of the SMC (**E**) to analyze LB formation in *Epm2b*^−/−^ mice 3 and 9 months after a single ICV injection. (**C**,**D**,**F**) Quantitative comparison of LB numbers in the CA1 (**C**) and CA2-CA3 (**D**) regions of the hippocampus, and in layers IV-V of the SMC (**F**), 3 and 9 months post-treatment. LB quantification in the SMC was conducted within the enlarged region (width: 747 px; height: 1550 px), corresponding to layers IV-V. Results are expressed as the median of independent samples, with boxplot bars indicating minimum and maximum values. Values were normalized using data from *Epm2b*^−/−^ mice injected with rAAV-Null as a reference. Statistical analysis was conducted using a non-parametric Mann–Whitney test. *n* = 4 mice per group. Scale bars = 25 µm in (**A**,**B**); 50 µm in (**E**).

PAS-D staining was used to identify LBs within the CA1 (Figure 2A,C) and CA2-CA3 (Figure 2B,D) regions of the hippocampus, and layers IV-V of the sensorimotor cortex (SMC) (Figure 2E,F). LBs were quantified 3 and 9 months following administration of either the rAAV-h*EPM2B* or rAAV-Null vectors. No therapeutic benefit was observed, since there was no reduction in the number of LBs in any of the ages or regions analyzed.

### 2.3. Treatment with rAAV-hEPM2B Partially Reduces Astrogliosis, Microgliosis, and Neuronal Loss in Epm2b^−/−^ Mice

**Figure 3:**
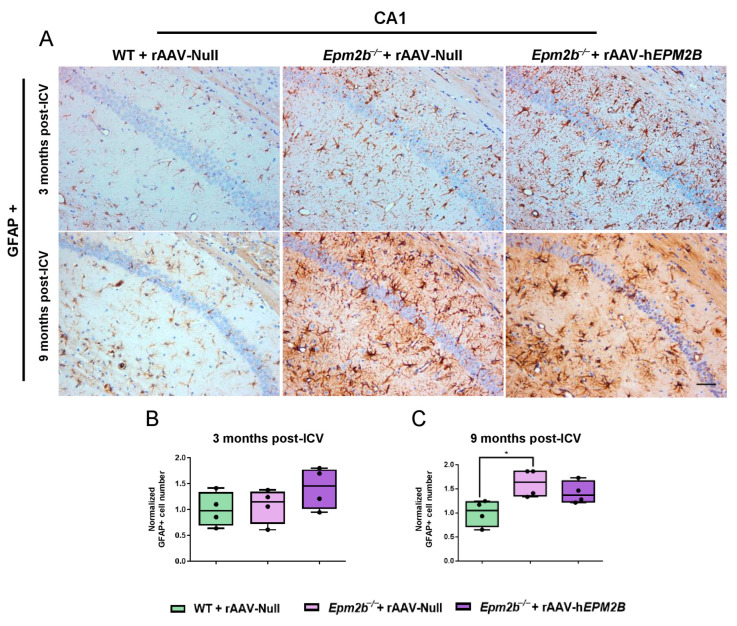
Analysis of astrogliosis in the CA1 region of the hippocampus in *Epm2b*^−/−^ mice 3 and 9 months post-injection: (**A**) Representative images of GFAP immunostaining in the CA1 field of the hippocampus 3 and 9 months after rAAV-h*EPM2B* or rAAV-Null administration in *Epm2b*^−/−^ mice. (**B**,**C**) Quantification of GFAP-positive cells expressed as the median of independent samples. Boxplot bars indicate minimum and maximum values. Data were normalized to levels observed in WT mice injected with rAAV-Null. Statistical analysis was conducted using a non-parametric Kruskal–Wallis test followed by Dunn’s multiple-comparison test. * *p* < 0.05. *n* = 4 mice per group. Scale bar = 50 µm.

**Figure 4:**
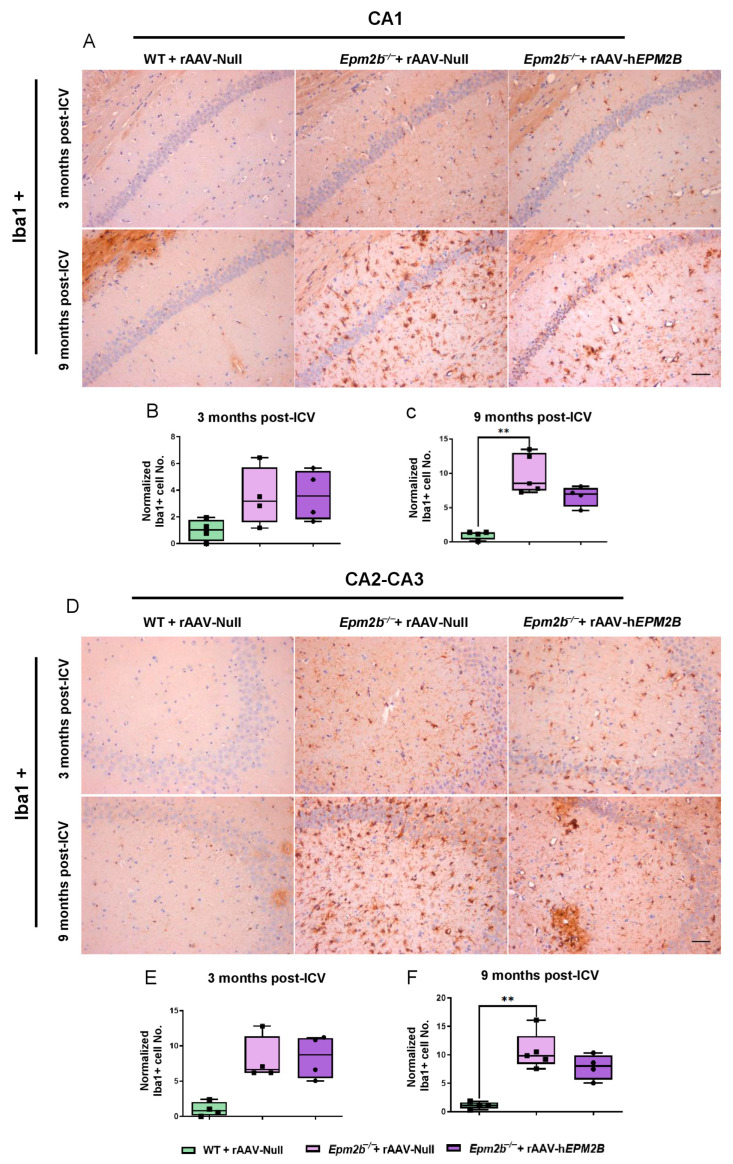
Microgliosis in the hippocampus of *Epm2b*^−/−^ mice: (**A**,**D**) IHC with an anti-Iba1 antibody to localize microglia. (**B**,**C**,**E**,**F**) Quantitative analysis of microgliosis in the CA1 (**B**,**C**) and CA2-CA3 (**E**,**F**) regions of the hippocampus of WT mice injected with rAAV-Null and *Epm2b*^−/−^ mice treated with rAAV-h*EPM2B* or rAAV-Null, 3 months (**B**,**E**) and 9 months (**C**,**F**) post-treatment. Results are expressed as the median of independent samples, with bars in the boxplots representing minimum and maximum values. Values were normalized to those of WT mice injected with rAAV-Null. Statistical analysis was performed using a non-parametric Kruskal–Wallis test followed by Dunn’s multiple-comparison test. ** *p* < 0.01. *n* = 4 mice per group. Scale bars = 50 µm.

To assess the effects of rAAV-h*EPM2B* administration on neuroinflammation and neurodegeneration in *Epm2b*^−/−^ mice, we quantified GFAP-, Iba1- and NeuN-positive cells in the CA1 (Figure 3, Figure 4A–C and Figure S2, respectively) and CA2-CA3 (Figure S1A–C, Figure 4D–F; Figure S3A–C; respectively) regions of the hippocampus, as well as in layers IV-V of the SMC (Figure S1D–F and Figure S3D–F, respectively). *Epm2b*^−/−^ mice treated with rAAV-Null displayed increased astrogliosis (measured as the presence of the GFAP marker) in the CA1 hippocampal region 9 months post-ICV injection, with significant differences compared to the control group (Figure 3). Treatment with the rAAV-h*EPM2B* vector showed a tendency to reduce astrogliosis in this region, reaching similar levels to those seen in WT mice 9 months post-treatment (Figure 3A,C). However, no significant differences were observed in other regions analyzed (CA2-CA3 region of the hippocampus and layers IV–V of the SMC) at either 3 or 9 months post-treatment (Figure S1).

We also analyzed microgliosis in the CA1 and CA2-CA3 regions of the hippocampus and in layers IV–V of the SMC in *Epm2b*^−/−^ mice 3 and 9 months post-injection (Figure 4). The hippocampus of *Epm2b*^−/−^ mice treated with the rAAV-h*EPM2B* vector displayed a reduction in microgliosis compared to those injected with the rAAV-Null vector, which exhibited a significant increase in microgliosis relative to the control group, 9 months after injection. This reduction was observed in both the CA1 (Figure 4A–C) and CA2-CA3 (Figure 4D–F) regions of the hippocampus. However, no significant differences were detected 3 months after treatment in any analyzed regions of the hippocampus (Figure 4B,E), nor in layers IV–V of the SMC either 3 or 9 months after ICV administration. 

Neuronal loss analysis in *Epm2b*^−/−^ mice, 3 and 9 months after rAAV-h*EPM2B* treatment, revealed a partial increase in NeuN-positive cells in the CA1 region of the hippocampus (Figure S2). However, despite this trend, the differences did not reach statistical significance (Figure S2B,C). No significant differences were observed between experimental groups in the CA2-CA3 region (Figure S3A–C) or in layers IV–V of the SMC (Figure S3D–F).

### 2.4. Treatment with rAAV-hEPM2B Reduces the Levels of Neuroinflammatory and Cell Death Markers in Epm2b^−/−^ Mice

**Figure 5:**
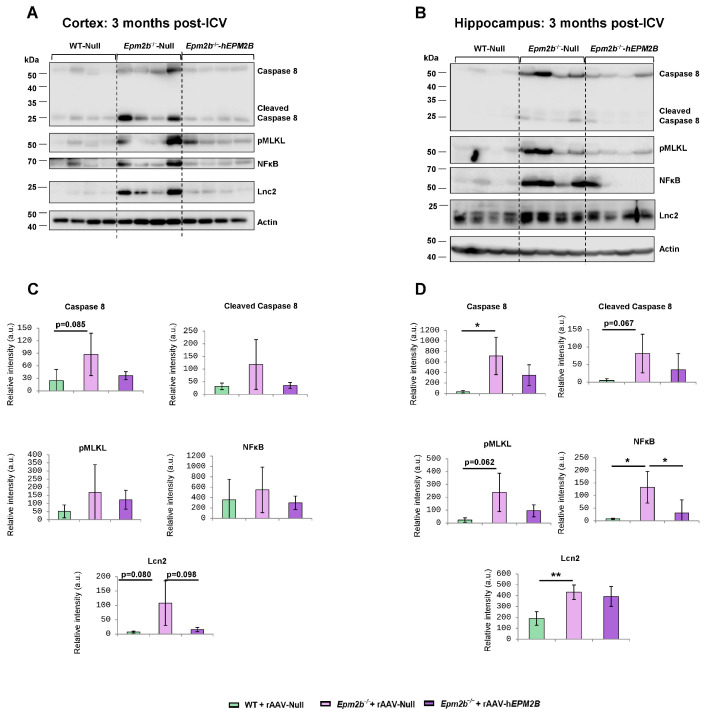
Levels of caspase-8 and NFkB in the cortex and hippocampus 3 months post-ICV injection of rAAV-Null or rAAV-h*EPM2B* in WT and *Epm2b*^−/−^ mice: (**A**–**D**) Protein levels of caspase-8 (full length and cleaved), NFκB, and actin assessed by Western blot in cortical (**A**,**C**) and hippocampal (**B**,**D**) extracts from WT mice injected with rAAV-Null and *Epm2b*^−/−^ mice treated with either rAAV-h*EPM2B* or rAAV-Null. Molecular weight standards are shown on the left. Densitometric quantification of the blots (**C**,**D**) was carried out as described in the Section 4, with values normalized to actin and represented as arbitrary units (a.u.). Four independent samples from each genotype were analyzed. Results are expressed as means +/− standard deviation (SD). Differences between paired samples were analyzed by two-tailed Student’s *t*-tests using GraphPad Prism version 5.0 statistical software (La Jolla, CA, USA). Statistical significance was considered at * *p* < 0.05 and ** *p* < 0.01.

Neuroinflammation has recently been identified as a significant and novel feature of LD, contributing to its pathogenesis and progression. To obtain a deeper understanding of the potential beneficial effects of rAAV-h*EPM2B* on various neuroinflammatory mediators in LD, we analyzed the levels of caspase-8, the phosphorylated form of the mixed lineage kinase domain-like (pMLKL) protein, NF-kB, chemokine CXCL10, and lipocalin 2 (Lcn2), as described in [47,48], in cortical and hippocampus extracts from WT mice injected with rAAV-Null and *Epm2b*^−/−^ mice treated with either rAAV-h*EPM2B* or rAAV-Null. Three and nine months post-injections, *Epm2b*^−/−^ mice treated with rAAV-Null exhibited increased levels of several neuroinflammatory markers in the cortex and, especially, in the hippocampus compared to WT mice (Figure 5 and Figure S4). Interestingly, *Epm2b*^−/−^ mice treated with rAAV-h*EPM2B* showed a reduction in some of these markers (Figure 5 and Figure S4). A significant decrease in NF-kB levels was observed in the hippocampus 3 months after ICV injection, with a clear trend toward reduced caspase-8, pMLKL, and Lcn2 expression, the latter particularly evident in the cortex (Figure 5). Notably, this trend was sustained up to 9 months post-treatment, although differences did not reach statistical significance (Figure S4). These findings suggest that rAAV-h*EPM2B* administration may exert a sustained modulatory effect on specific components of the neuroinflammatory response, especially in the hippocampus.

### 2.5. rAAV-hEPM2B-Based Gene Therapy Reverses Episodic Memory Alterations and Improves Spontaneous Locomotor Activity but Does Not Correct Motor Coordination Impairments in Epm2b^−/−^ Mice

**Figure 6:**
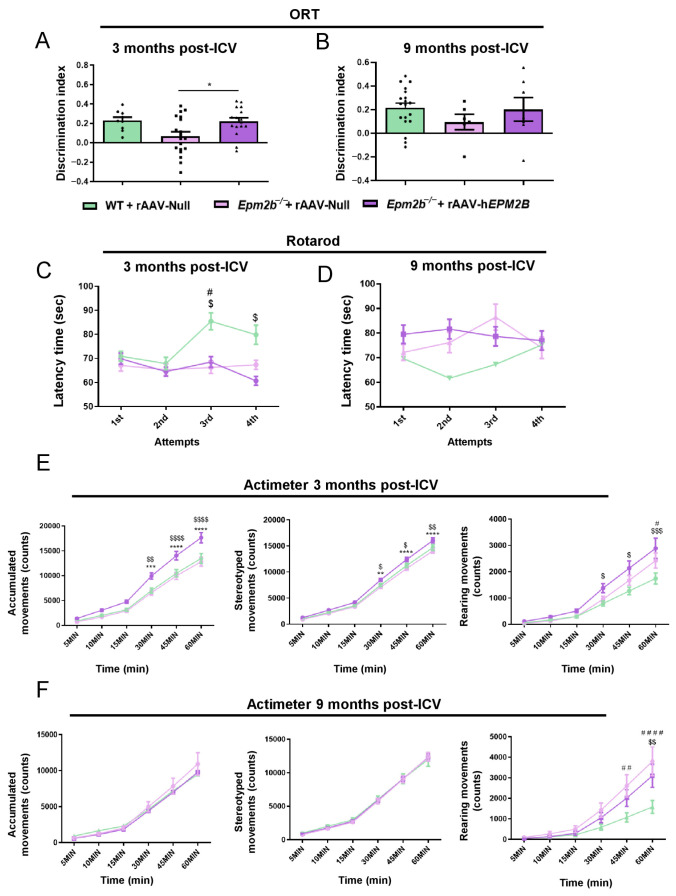
Behavioral assessments in *Epm2b*^−/−^ mice treated with rAAV-h*EPM2B*, and in *Epm2b*^−/−^ and WT mice injected with rAAV-Null: (**A**,**B**) Memory performance based on the D.I. obtained from the ORT, 3 (**A**) and 9 (**B**) months post-ICV injection. (**C**,**D**) Evaluation of motor coordination based on the latency to fall from the cylinder in the rotarod test, 3 (**C**) and 9 (**D**) months post-ICV treatment (# WT + rAAV-Null vs. *Epm2b*^−/−^ + rAAV-Null; $ WT + rAAV-Null vs. *Epm2b*^−/−^ + rAAV-h*EPM2B*). (**E**,**F**) Analysis of spontaneous accumulated, stereotyped, and rearing movements recorded over one hour in the actimeter, 3 (**E**) and 9 (**F**) months after ICV injection (* *Epm2b*^−/−^ + rAAV-h*EPM2B* vs. *Epm2b*^−/−^ + rAAV-Null; # WT + rAAV-Null vs. *Epm2b*^−/−^ + rAAV-Null; $ WT + rAAV-Null vs. *Epm2b-/-* + rAAV-h*EPM2B*). Data are shown as mean ± SEM. Statistical comparison between groups was conducted using one- and two-way ANOVA with Tukey’s multiple-comparison tests. * *p* < 0.05, ** *p* < 0.01, *** *p* < 0.001, **** *p* < 0.0001. *n* = 15–25 mice per group per experiment.

Object recognition task (ORT) was performed to evaluate the episodic memory state in *Epm2b*^−/−^ mice 3 and 9 months after the administration of rAAV-h*EPM2B* or rAAV-Null vectors (Figure 6A,B). Memory performance was significantly improved in *Epm2b*^−/−^ mice 3 months after treatment to levels comparable to WT mice, as indicated by the recovery of the D.I. between the old and new object (Figure 6A). However, 9 months after treatment (Figure 6B), the differences between WT and *Epm2b*^−/−^ mice injected with the rAAV-Null or rAAV-h*EPM2B* vectors were not statistically significant. Despite this, *Epm2b*^−/−^ mice treated with the rAAV-h*EPM2B* vector showed a tendency to recover the mild memory deficits present in aged *Epm2b*^−/−^ mice (Figure 6B).

Assessment of motor coordination with the rotarod test revealed that *Epm2b*^−/−^ mice treated with rAAV-h*EPM2B* or rAAV-Null vectors remained on the rod for a shorter period than WT mice 3 months after injection (Figure 6C), although no differences were found 9 months after treatment administration (Figure 6D).

Examination of spontaneous motor activity showed that rAAV-h*EPM2B* treatment led to an improvement in spontaneous accumulated, stereotyped, and rearing movements 3 months post-injection (Figure 6E). *Epm2b*^−/−^ treated mice presented higher spontaneous locomotor activity than WT control mice, indicating some level of hyperactivity (Figure 6E). However, 9 months after treatment, *Epm2b*^−/−^ mice treated with rAAV-hEPM2B showed no differences in locomotor activity in comparison to rAAV-Null treated mice (Figure 6F).

### 2.6. Treatment with rAAV-hEPM2B in Epm2b^−/−^ Mice Does Not Improve Spontaneous Electrical Brain Activity or Reduce PTZ Sensitivity

**Figure 7:**
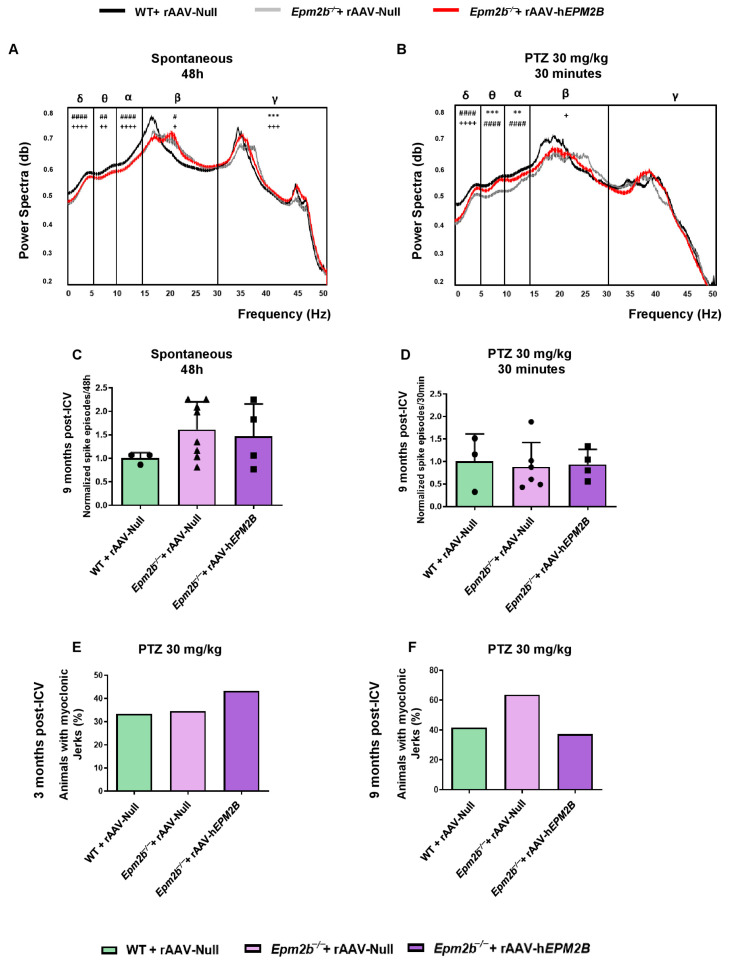
EEG and PTZ susceptibility analysis in WT and *Epm2b*^−/−^ mice 3 and 9 months post-ICV injection of rAAV-h*EPM2B* or rAAV-Null vectors: (**A**) Representative normalized power spectra from baseline EEG recordings over a 48 h period. (**B**) Representative normalized power spectra from EEG recordings after IP injection of subconvulsive doses of PTZ (30 mg/kg). Data are presented as mean ± SEM. A one-way ANOVA with Tukey’s multiple-comparison test was performed on the areas under the curve obtained by plotting EEG powers across all experimental groups. ** *p* < 0.01, *** *p* < 0.001; + *p* < 0.05, ++ *p* < 0.01, +++ *p* < 0.001, ++++ *p* < 0.0001; # *p* < 0.05, ## *p* < 0.01, #### *p* < 0.0001 (+ WT + rAAV-Null vs. *Epm2b*^−/−^ + rAAV-h*EPM2B*; # WT + rAAV-Null vs. *Epm2b*^−/−^ + rAAV-Null; *Epm2b*^−/−^ + rAAV-h*EPM2B* vs. *Epm2b*^−/−^ + rAAV-Null). *n* = 3–6 mice per group. (**C**) Spontaneous interictal spikes and (**D**) PTZ-induced interictal spikes in WT and *Epm2b*^−/−^ mice 9 months after ICV administration of rAAV-h*EPM2B* or rAAV-Null. A non-parametric Kruskal–Wallis test followed by Dunn’s multiple-comparison test was used. Data are shown as normalized mean ± SEM, with values normalized to those of WT mice injected with rAAV-Null values. *n* = 3–8 mice per group. (**E**,**F**) Percentage of animals displaying myoclonic jerks after IP injection of 30 mg/kg PTZ in WT and *Epm2b*^−/−^ mice 3 (**E**) or 9 (**F**) months after ICV injection. Data are shown as percentages, with Fisher’s exact test applied between experimental groups. *n* = 15–25 mice per group per experiment.

To analyze epileptic-like activity, we performed video-EEG recordings 9 months after treatment (Figure 7A,B). Analysis of basal activity rhythms revealed an altered EEG in *Epm2b*^−/−^ mice injected with rAAV-Null or rAAV-h*EPM2B* vectors (Figure 7A). After intraperitoneal injection of 30 mg/kg PTZ, power spectra were decreased in all experimental groups, possibly reflecting a desynchronization of neuronal firing induced by the compound (Figure 7B). Both *Epm2b*^−/−^ mice treated with rAAV-h*EPM2B* or rAAV-Null showed a significantly decreased power spectrum of delta low-frequency waves. Notably, gene therapy normalized power spectra in both theta and alpha frequency waves after PTZ injection; however, only *Epm2b*^−/−^ mice treated with the rAAV-h*EPM2B* vector exhibited lower beta wave power spectra (Figure 7B). These findings suggest a differential impact of gene therapy on specific frequency bands.

*Epm2b*^−/−^ mice treated with rAAV-h*EPM2B* or rAAV-Null exhibited a similar amount of spontaneous interictal spikes compared to WT mice (Figure 7C). Furthermore, after administration of 30 mg/kg PTZ, all groups displayed a comparable amount of interictal spikes (Figure 7D). The same percentage of mice displayed myoclonic jerks 3 (Figure 7E) and 9 (Figure 7F) months after rAAV-h*EPM2B* or rAAV-Null injections.

## 3. Discussion

Gene therapy-based strategies are considered promising therapeutic approaches for monogenic diseases. We recently reported a gene therapy approach aimed at inducing laforin expression in the *Epm2a*^−/−^ mouse model using a rAAV vector containing the coding region of the human *EPM2A* gene. A single ICV injection of this vector resulted in significant neurological improvements [46]. In the present study, we demonstrate that restoring *EPM2B* gene expression with a rAAV vector in the *Epm2b*^−/−^ mouse model reverses some neurological impairments, although the therapeutic effects were less pronounced compared to restoring laforin function in *Epm2b*^−/−^ mice [46].

RT-qPCR analysis of brain samples revealed that ICV administration of the rAAV-h*EPM2B* vector achieved *EPM2B* transgene expression levels in *Epm2b*^−/−^ mice comparable to endogenous *Epm2b* expression in WT controls. Due to the lack of reliable malin-specific antibodies, we used a rAAV2/9 vector carrying the *GFP* reporter gene under the same CAG promoter to assess vector biodistribution throughout the central nervous system. Consistent with our previous findings [46], the rAAV-*GFP* vector transduced the hippocampus, cortex, and fimbria. Based on these results, we assumed a similar distribution pattern for malin expression.

We evaluated the effect of a single ICV injection of the rAAV-h*EPM2B* vector on LB formation in *Epm2b*^−/−^ mice at 3 and 9 months post-treatment. Unlike the recovery of the *hEPM2A* gene expression in *Epm2a*^−/−^ mice [46], restoration of h*EPM2B* gene expression in *Epm2b*^−/−^ mice did not reduce LB accumulation. These findings contrast with a recent report showing beneficial effects of restoring malin function in *Epm2b*^−/−^ mice containing an inducible malin expression cassette [49]. In that study, the authors showed that although malin expression did not degrade insoluble glycogen or remove preexisting LBs, it prevented further LB accumulation [49]. The lack of LB reduction observed in our study may be attributed to insufficient h*EPM2B* expression. As noted above, RT-qPCR analyses confirmed transgene expression; however, the malin protein may not be as stable as its endogenous murine counterpart.

The formation of a functional laforin-malin complex is essential for recruiting malin to glycogen particles and regulating glycogen synthesis [16]. It has been shown that brains from *Epm2b*^−/−^ mice exhibit increased levels of laforin in the insoluble fraction of brain extracts compared to WT mice [19,31,50], a fraction that also displays a marked increase in insoluble glycogen [19]. This elevated laforin content likely results from the absence of malin-dependent ubiquitination. These results suggest that laforin, found in the LBs of *Epm2b*^−/−^ mice [51], may be bound to insoluble glycogen, thereby rendering it inactive or functionally impaired. The expressed malin protein may therefore not fully interact with endogenous murine laforin, resulting in an incomplete or non-functional complex. Unfortunately, the lack of a reliable antibody to detect malin expression hinders our ability to further explore that option. Importantly, in a recent study, we administered an rAAV-based malin gene therapy systemically at a presymptomatic stage (1 month old) and observed a clear reduction in LB formation. These findings strongly support our hypothesis and highlight that treatment timing is a key determinant of therapeutic success [52].

Treatment with the rAAV-h*EPM2B* vector in *Epm2b*^−/−^ mice reduces neuroinflammation, as evidenced by decreased levels of GFAP, Iba1, and different pro-inflammatory mediators. These results are consistent with previous findings obtained following malin recovery [49]. Despite a direct relationship between polyglucosan accumulation and neuroinflammation [53], malin also modulates inflammatory pathways independently of the laforin-malin complex [48,54,55]. Our results show that restoring malin function in *Epm2b*^−/−^ mice reduces neuroinflammation, thereby improving specific behavioral impairments, including defects in episodic memory and spontaneous locomotor activity. Although treated mice display higher locomotor activity than WT controls, this increase is interpreted as a functional improvement consistent with other molecular and behavioral corrections. Nevertheless, it should be noted that partial restoration could theoretically result in a new, non-physiological behavioral state.

Seizures represent one of the most severe symptoms and can result in heightened morbidity and mortality in LD patients. We evaluated the efficacy of a single ICV injection of the rAAV-h*EPM2B* vector on epileptic activity in *Epm2b*^−/−^ mice 9 months after treatment administration. Video-EEG and PTZ sensitivity tests showed that rAAV-h*EPM2B*-based gene therapy did not restore normal brain electrical activity or normal neuronal excitability. This lack of antiepileptic benefit of the rAAV-h*EPM2B* vector could be associated with the inability of malin to form a functional complex with laforin, which regulates the abundance of several proteins involved in neuronal excitability [46]. The absence of this complex not only leads to LB accumulation but may also disrupt additional laforin–malin–dependent regulatory mechanisms beyond glycogen metabolism, both of which could contribute to neuronal hyperexcitability and seizure susceptibility [25,43,46,56,57,58,59,60].

In conclusion, our work demonstrates that rAAV-based gene therapy, inducing malin expression with the coding region of the human *EPM2B* gene, produces relevant therapeutic benefits in the *Epm2b*^−/−^ mouse model. However, its efficacy is lower than that observed following laforin restoration in the *Epm2a*^−/−^ mouse model. Further studies and alternative strategies are required to elucidate the mechanisms underlying this divergent therapeutic response and to develop an effective therapy for LD patients carrying *EPM2B* mutations.

## 4. Materials and Methods

### 4.1. Experimental Animals

The colonies of *Epm2b*^−/−^ and WT mice were housed in isolated cages within a controlled environment at the Animal Facility Service of the Instituto de Investigación Sanitaria-Fundación Jiménez Díaz. The conditions included a 12:12 light/dark cycle, a constant temperature of 23 °C, and ad libitum access to food and water. Animal welfare was prioritized throughout the experiments, with efforts made to minimize distress and to use the minimum number of animals necessary for sacrifice. Since no gender-related phenotypic differences have been described in mice or patients with LD, data from male and female mice were analyzed interchangeably. All procedures were carried out in accordance with the ARRIVE guidelines [61], as well as the ethical standards set by the Instituto de Investigación Sanitaria-Fundación Jiménez Díaz Ethical Review Board, the European Communities Council Directive (2010/63/EU), and the “Principles of Laboratory Animal Care” (NIH publication No. 86–23, updated 1985).

### 4.2. Production of rAAV2/9-CAG-hEPM2B, rAAV2/9-CAG-Null and rAAV2/9-CAG-GFP Vectors

The rAAV2/9-CAG-h*EPM2B* (rAAV-h*EPM2B*) vector, carrying the cDNA of the h*EPM2B* gene (NM_198586.3), the rAAV2/9-CAG-Null (rAAV-Null) vector, and the rAAV2/9-CAG-GFP (rAAV-*GFP*) vector were produced at the Unitat de Producció de Vectors (UPV_ www.viralvector.eu, accessed on 2 March 2021). These vectors were generated following the triple transfection system: (a) the ITR-containing plasmid; (b) the plasmid encoding the AAV capsid proteins (VP1, VP2, and VP3) and replicate genes; and (c) the adenoviral helper plasmid. AAV vectors were purified using iodixanol-based ultracentrifugation to remove empty capsids [62].

### 4.3. Stereotaxic ICV Injections

Stereotaxic ICV injections were performed at 3 months of age, as previously described [46]. *Epm2b*^−/−^ and WT mice received a single ICV injection of either rAAV-h*EPM2B*, rAAV-Null, or rAAV-*GFP* vectors. To induce anesthesia, 4% isoflurane and 2% oxygen were administered, while 2% isoflurane and 1.5% oxygen were used to maintain it. The mice were secured in a stereotaxic frame (Stoelting, Wood Dale, Illinois, USA). To ensure hydration, a subcutaneous saline injection (1 mL) was given, and ophthalmic gel was applied to prevent dry eyes. A heating pad was used to maintain a body temperature of 37 °C. After sterilizing the area with 70% ethanol, a 2 cm incision was made behind the eyes. H_2_O_2_ was used to mark the bregma and lambda spots on the skull. A small burr hole was drilled using the coordinates of the right cerebral lateral ventricle (anteroposterior −0.3 mm, mediolateral −0.9 mm, and dorsoventral −2.5 mm). Then, using a Hamilton^®^ syringe (ThermoFisher Scientific, Waltham, MA, USA, Cat. #10664301), 3 µL of viral suspension (titer: 1.26 × 10^12^ vg/mL) was administered at a rate of 1 µL/min. After suturing the incision, analgesia was provided by administering 5 mg/kg of meloxicam (Boehringer Ingelheim, Ingelheim, Germany). A total of 40 mice were used in each group and condition.

### 4.4. RNA Extraction and Quantitative Reverse Transcription-Polymerase Chain Reaction

Brain tissues were homogenized on ice using TRIzolTM Reagent (ThermoFisher Scientific, Waltham, MA, USA) to extract RNA. The extracted RNA pellets were cleaned, dried, and resuspended before applying DNase Enzyme (ThermoFisher Scientific, Waltham, MA, USA) to remove any DNA traces. Quantification was carried out using a NanoDrop ND-1000 spectrophotometer (ThermoFisher, Waltham, MA, USA). For RT-PCR, 1 µg of RNA per reaction was used with the High-Capacity cDNA Reverse Transcription Kit with RNase inhibitor (ThermoFisher Scientific, Waltham, MA, USA). RT-PCR settings were as follows: 25 °C for 10 min, 37 °C for 120 min, and 85 °C for 5 min. The resulting h*EPM2B* cDNA was used as a template for quantitative RT-PCR (RT-qPCR), performed using TaqMan™ Fast Advanced Master Mix (ThermoFisher Scientific, Waltham, MA, USA). Probes used included *Epm2b* (ThermoFisher Scientific, Waltham, MA, USA; Mm00614667_s1; Cat: #4331182), *EPM2B* (ThermoFisher Scientific, Waltham, MA, USA; Hs01112790_s1; Cat: #4331182), and *Gapdh* (ThermoFisher Scientific, Waltham, MA, USA; Mm99999915_g1; Cat: #4448491). qPCR conditions were: 50 °C for 2 min, 95 °C for 2 min, followed by 40 cycles of 95 °C for 1 sec and 60 °C for 20 sec. Triplicates of each sample were analyzed, and results were calculated using the 2^−ΔΔCT^ method.

### 4.5. PAS-Diastase Staining and Immunohistochemistry

PAS-diastase (PAS-D), immunohistochemistry (IHC), and immunofluorescence in paraffin (IF-P) procedures were performed following the protocol described in [46,63]. Sections were rehydrated with decreasing concentrations of alcohol. For PAS-D staining, they were treated with porcine pancreas α-amylase (5 mg/mL in dH2O) (Merck, Darmstadt, Germany), processed with the PAS Kit (Merck, Darmstadt, Germany), and counterstained with Gill No. 3 hematoxylin (Merck, Darmstadt, Germany). For IHC, rehydrated sections were subjected to antigen retrieval by boiling in 0.1 M sodium citrate buffer, pH 6.0. Samples were then incubated in blocking buffer (1% bovine serum albumin, 5% fetal bovine serum, 2% Triton X-100, diluted in PBS), followed by incubation with primary antibodies diluted in the same blocking buffer. The primary antibodies used were green fluorescent protein (GFP) (1:100 dilution; Abcam, Cambridge, UK; Cat. #ab183734), Neuronal nuclei (NeuN) (1:100 dilution; Millipore, Temecula, CA, USA; Cat. # MAB377), ionized calcium-binding adapter molecule 1 (Iba1) (1:100 dilution; ThermoFisher Scientific, MA, USA; #MA536257), and glial fibrillary acidic protein (GFAP) (1:1000 dilution; Millipore, Temecula, CA, USA; Cat. #MAB360). After incubation, sections were treated with biotinylated secondary antibodies, stained with the Vectastain ABC kit (Vector Laboratories, Burlingame, CA, USA), visualized with diaminobenzidine (DakoCytomation, Glostrup, Denmark) and H_2_O_2_, and counterstained with Carazzi hematoxylin (Panreac Quimica, Barcelona, Spain).

Two consecutive sections per animal from 4–6 mice were analyzed in the CA1 and CA2-CA3 regions of the hippocampus and layers IV-V of the SMC. Immunoreactivity was quantified by two researchers using ImageJ 1.54g software (NIH, Bethesda, MD, USA), with results reported as the means of both quantifications.

### 4.6. Western Blot Analyses

Samples from the mouse brain (hippocampus and cortex) were lysed in RIPA buffer [50 mM Tris-HCl, pH 8; 150 mM NaCl; 0.5% sodium deoxycholate; 0.1% SDS; 1% Nonidet P40; 1mM PMSF; and a complete protease inhibitor cocktail (Roche, Barcelona, Spain)] for 30 min at 4 °C with occasional vortexing. The lysates were passed ten times through a 25-gauge needle in a 1 mL syringe and centrifuged at 13,000× *g* for 15 min at 4 °C. Supernatants were collected, and 35 μg protein was subjected to SDS-PAGE and transferred onto a PVDF membrane. Membranes were blocked in 5% (*w*/*v*) nonfat milk in Tris-buffered saline (TBS-T: 50 mM Tris-HCl, 150 mM NaCl, pH 7.4; with 0.1% Tween-20) for 1 h at room temperature and then incubated overnight at 4 °C with the corresponding primary antibodies: anti-NF-kB (Santa Cruz Biotechnologies, Dallas, TX, USA, Sc-8008), anti-caspase-8 (Cell Signaling, Danvers, MA, USA, 9746), anti-phospho (Ser345)-MLKL (Millipore, Burlington, Massachusetts, USA, MABC1158), anti-CXCL10 (Abcam, Cambridge, UK, ab9938) and anti-Lipocalin2 (Abcam, Cambridge, UK, ab63929). Anti-Actin (Sigma-Aldrich, St. Louis, MO, USA, A2066) was used as a housekeeping antibody. Membranes were then probed with suitable secondary antibodies for 1 h at room temperature. Signals were detected using chemiluminescence with ECL Prime Western Blotting Detection Reagents (Cytiva-Amersham, Marlborough, MA, USA, RPN2232) and imaged using the Fuji-LAS-4000 system (GE Healthcare, Barcelona, Spain). The results were analyzed using the software Image Studio Lite version 5.2 (LI-COR Biosciences, Bad Homburg, Germany). Experiments were performed on at least three individuals from each genotype, including both males and females.

### 4.7. Object Recognition Task

The ORT was used to analyze episodic memory. It involved habituating mice to a dark open field box for 10 min. After a 2 h break, two identical objects, A and B, were placed in the center of the box. The mice were allowed to explore both objects freely, and the time spent examining each object (tA and tB) was recorded. The same test was repeated 2 h later, but object B was replaced with a new object, C. The mice’s exploration times (tA and tC) were measured using virtual timers generated by XNote Stopwatch 1.68 software, which were activated when the mice inspected the object from a distance of 2 cm or less. The discrimination index (D.I.) was calculated using the following formula: D.I. = (tC − tA)/(tC + tA) [63].

### 4.8. Motor Coordination

To evaluate the balance and motor coordination of mice, the rotarod test (Harvard Apparatus, Holliston, MA, USA) was used. After two days of training, only those mice that could stay on the cylinder for at least 60 s were included in the analysis. The latency time to fall from the rotating cylinder was measured twice a day, with each session lasting a maximum of five minutes, as the speed increased from 4 rpm to 40 rpm [63].

### 4.9. Spontaneous Locomotor Activity

Spontaneous movements of mice were monitored using a computerized actimeter (Harvard Apparatus, Holliston, MA, USA). The device detects breaks in an infrared light beam and records mouse crossings in an open field. SEDACOM 1.4 software (Harvard Apparatus, Holliston, MA, USA) was used to analyze spontaneous, rearing, and stereotyped movements at 5, 10, 15, 30, 45, and 60 min [63].

### 4.10. Video-EEG Recording

A plastic pedestal with trimmed electrodes (Plastics1, Roanoke, VA, USA) was surgically implanted onto the skull and secured with acrylic resin. To alleviate post-surgical pain, meloxicam (5 mg/kg) (Boehringer Ingelheim, Ingelheim, Germany) was administered to the animals. Following the surgery, mice were given one week to recover before video-EEG testing began. The recordings were performed using a wireless transmitter (Epoch, BIOPAC Systems, Inc., Goleta, CA, USA) attached to the pedestal, and data were digitally recorded on a computer under free-motion conditions. Mice were observed in their cages for 2 days under basal conditions. Afterwards, they were recorded for 30 min following PTZ injections. The recordings were done at a sampling rate of 250 Hz, and a 50 Hz notch filter was applied. Digital video cameras were used to capture their behavior. The EEG data were analyzed both automatically and manually using Acknowledge^®^ 5.0 software (Epoch, BIOPAC Systems, Inc., Goleta, CA, USA). Any signal loss or artifacts were identified and excluded. Power spectra were calculated using a spectral estimator derived from autoregressive processes, ensuring normalized amplitudes for consistent peak-to-peak ranges. The Comb Band Stop filter and a Blackman window were then applied. Automated seizure analysis was conducted, which included monitoring interictal epileptiform discharges (IEDs) and seizures [46,63].

### 4.11. Sensitivity to PTZ

PTZ (Merck, Darmstadt, Germany) was administered intraperitoneally to mice at subconvulsive doses (30 mg/kg). This treatment was used to assess the percentage of mice exhibiting myoclonic jerks. The study lasted for 45 min, and each animal was examined by two different researchers [63].

### 4.12. Statistical Analysis

For behavioral tests, PTZ-based studies, and electrophysiology, a minimum of 12 mice per genotype were examined. For video-EEG and RT-qPCR assays, 4–6 mice per genotype were used. For immunohistochemistry and Western blot experiments, 4–8 mice per genotype were analyzed. The values are presented as the mean ± standard error of the mean (SEM), mean ± standard deviation (SD), or as percentages. To assess differences between experimental groups, one- or two-way ANOVA, Fisher’s exact test, the non-parametric Kruskal–Wallis test, or the Mann–Whitney test were used, as appropriate for each specific case. For EEG analysis, the area under the curve (AUC) was calculated to compare differences in the power spectra between groups. All statistical analyses were conducted using GraphPad Prism 8.0 (San Diego, CA, USA). All statistical tests were two-tailed, and the statistical significance thresholds were set at * *p* < 0.05, ** *p* < 0.01, *** *p* < 0.001, and **** *p* < 0.0001.

## Data Availability

The original contributions presented in this study are included in the article/Supplementary Materials. Further inquiries can be directed to the corresponding authors.

## Supplementary Materials

The following supporting information can be downloaded at: https://www.mdpi.com/article/10.3390/ijms262411930/s1.
