# Supplementary material for: Adeno-Associated Virus-Based Gene Therapy for Lafora Disease in Epm2b-Deficient Mice
Source: Int J Mol Sci. 2025 Dec 11;26(24):11930. doi: 10.3390/ijms262411930 (PMC12732665; doi:10.3390/ijms262411930)
Supplement: Supplementary file 1 [file ijms-26-11930-s001.zip › ijms-3985902-supplementary.pdf]

Article

Adeno-associated virus-based gene therapy for Lafora disease in *Epm2b*-deficient mice

Luis Zafra-Puerta[1,2], Nerea Iglesias-Cabeza[1], Pascual Sanz[3,4], María Adelaida García-Gimeno[5], Gema Sánchez-Martín[1], Marina P. Sánchez[1*], José M. Serratosa[1*]

1 Laboratory of Neurology, Instituto de Investigación Sanitaria-Fundación Jiménez Díaz, Universidad Autónoma de Madrid (IIS-FJD, UAM), 28040 Madrid, Spain
2 Fondazione Malattie Rare Mauro Baschirotto BIRD Onlus, Longare (VI), Italy
3 Instituto de Biomedicina de Valencia, CSIC, Jaime Roig 11, 46010-Valencia, Spain
4 Centro de Investigación Biomédica en Red de Enfermedades Raras (CIBERER), 46010-Valencia, Spain
5 Department of Biotechnology. Escuela Técnica Superior de Ingeniería Agronómica y del Medio Natural. Universitat Politécnica de València. 46022-Valencia, Spain
* Correspondence: JMSerratosa@fjd.es (JMS); Msanchezg@fjd.es (MPS) Tel.: (optional; include country code; if there are multiple corresponding authors, add author initials)

Supplementary Figures

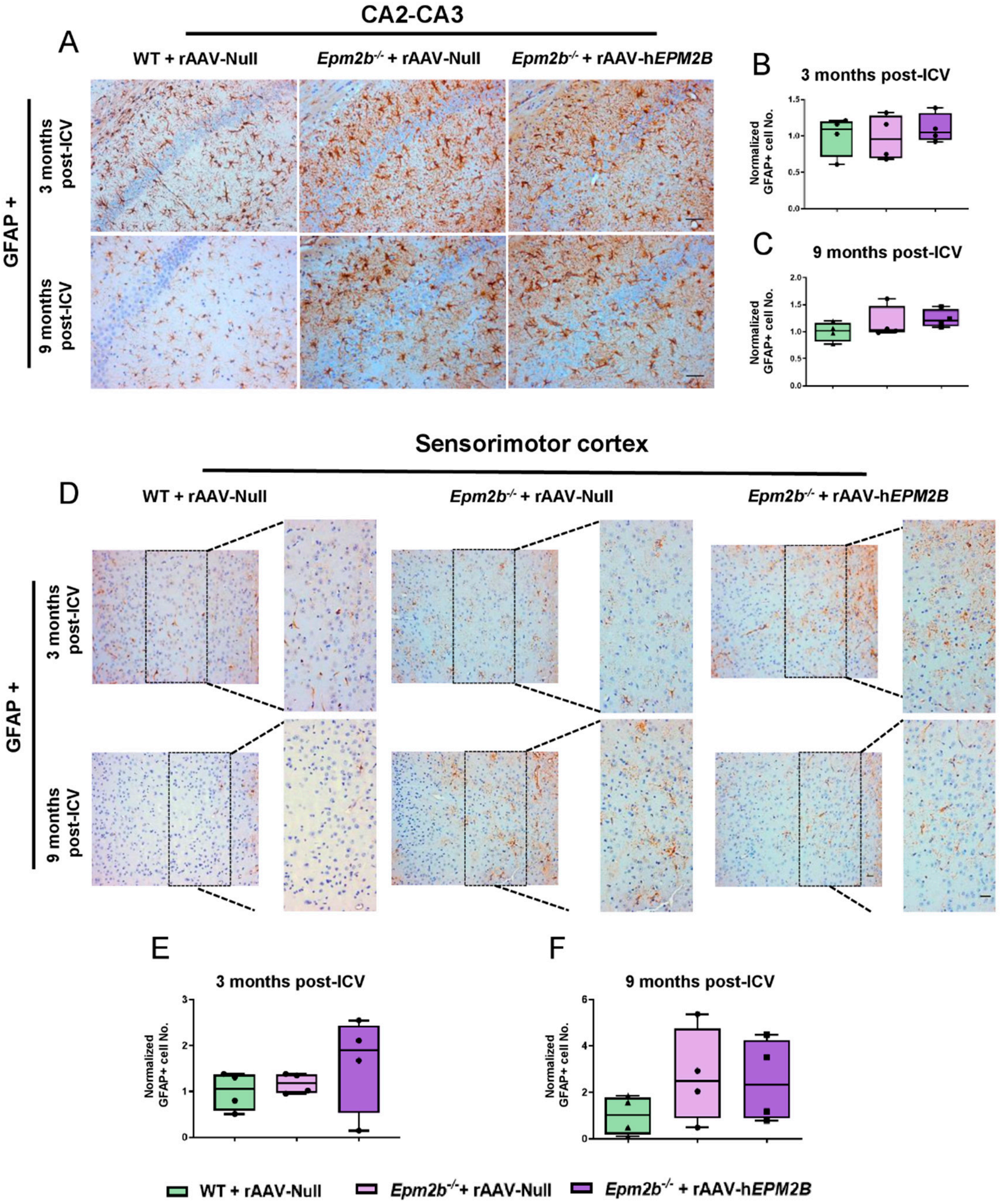

Supplemental Figure S1. Astrogliosis in the CA2-CA3 region of the hippocampus and layers IV-V of the SMC in *Epm2b*$^{-/-}$ mice: (A, D) IHC with an anti-GFAP antibody was conducted in WT mice injected with rAAV-Null and *Epm2b*$^{-/-}$ mice treated with rAAV-h*EPM2B* or rAAV-Null, 3 and 9 months post-ICV injection. (B, C, E, and F) Quantification of reactive astrocytes was performed in the CA2-CA3 region of the hippocampus (B, C) and layers IV-V of the SMC (E-F) at 3 and 9 months after treatment administration. In the SMC, the enlarged region (width: 747 px; height: 1550 px), corresponding to layers IV-V, was quantified.

Results are expressed as the median of independent samples. Boxplot bars show the minimum and maximum values. Values were normalized to levels from WT mice injected with rAAV-Null. Statistical analysis was conducted using a non-parametric Kruskal–Wallis test followed by Dunn's multiple-comparison test. n= 4 mice per group. Scale bars = 25μm in (A, B); 50μm in (E).

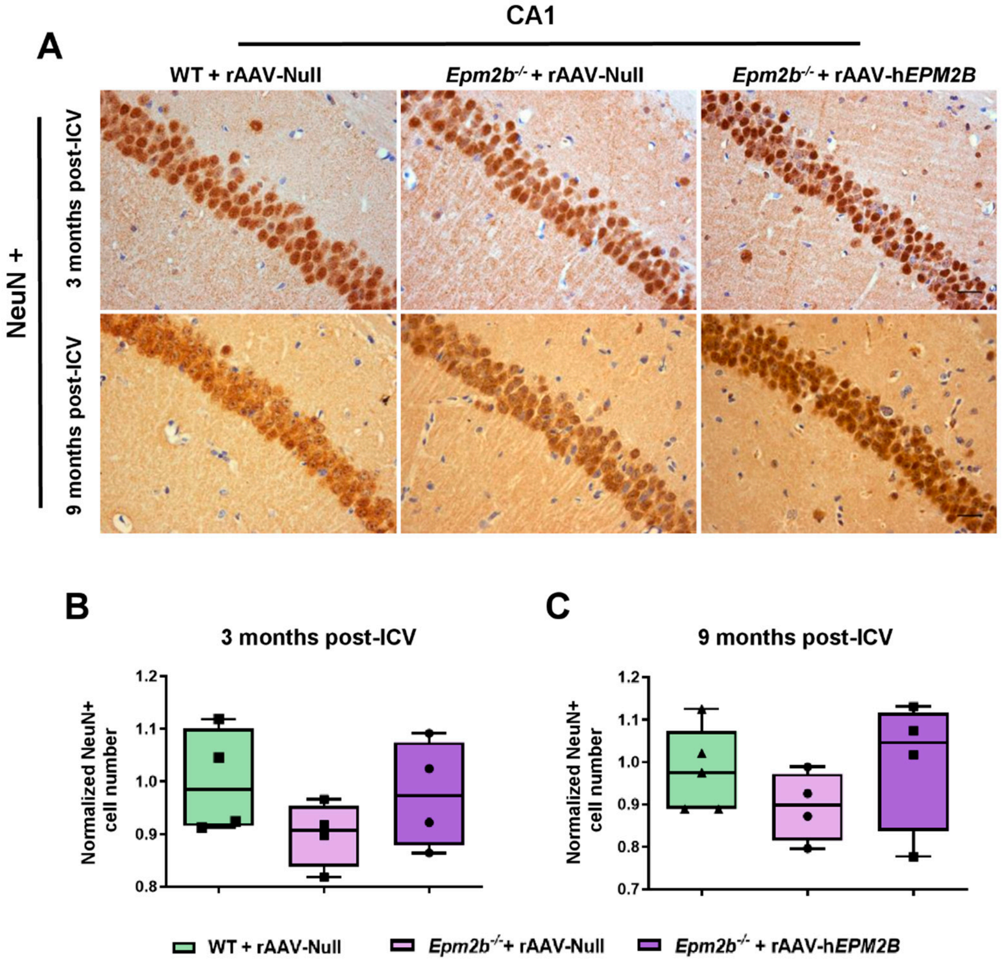

Supplemental Figure S2. Effect of rAAV-h*EPM2B* treatment on neurodegeneration in *Epm2b*$^{-/-}$ mice 3 and 9 months post-injection: (A) NeuN immunostaining in the CA1 region of the hippocampus of *Epm2b*$^{-/-}$ mice. (B, C) Quantification of NeuN-positive cells in the CA1 region of the hippocampus 3 (B) and 9 (C) months post-treatment. Results are expressed as the median of independent samples, with bars in the boxplots representing minimum and maximum values. Values were normalized to those of WT mice injected with rAAV-Null. Statistical analysis was performed using a non-parametric Kruskal–Wallis test followed by Dunn's multiple-comparison test. n = 4-5 mice per group. Scale bar = 50   m.

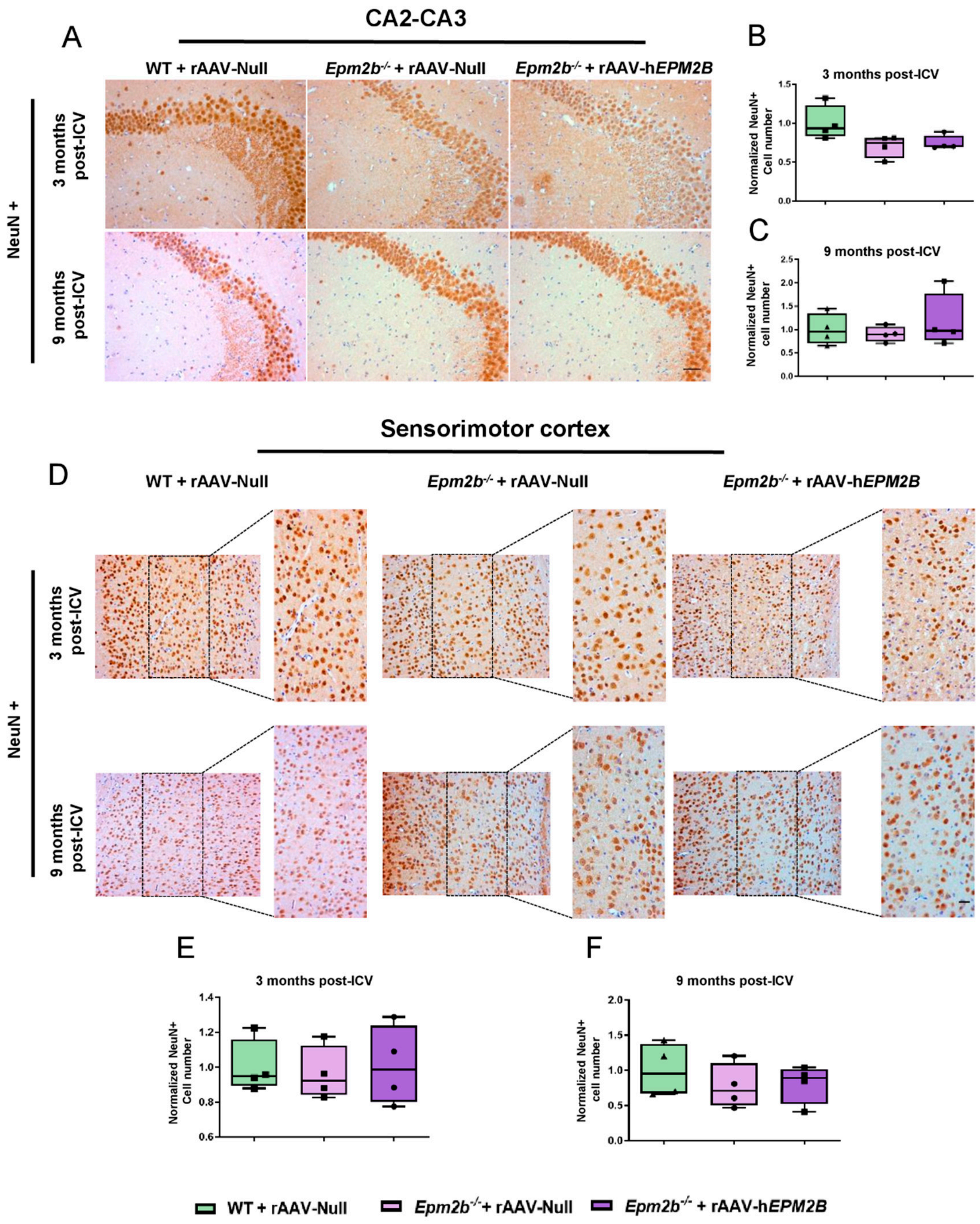

Supplemental Figure S3. NeuN immunostaining in the CA2-CA3 region of the hippocampus and layers IV-V of the SMC in *Epm2b*$^{-/-}$ mice: (A, D) IHC with the NeuN antibody in WT mice injected with rAAV-Null and *Epm2b*$^{-/-}$ mice treated with rAAV-h*EPM2B* or rAAV-Null, 3 and 9 months post-ICV injection. (B, C, E and F) Quantification of NeuN-positive cells in the CA2-CA3 region of the hippocampus (B, C) and layers IV-V of the SMC (E, F) 3 and 9 months after treatment. In the SMC, the enlarged region (width: 747 px; height: 1550 px), corresponding to layers IV-V, was quantified. Results are expressed as the median of independent samples, with boxplot bars showing minimum and maximum values. Values were normalized

to those of WT mice injected with rAAV-Null. Statistical analysis was conducted using a non-parametric Kruskal–Wallis test followed by Dunn's multiple-comparison test. n = 4 mice per group. Scale bars = 25µm in (A, B); 50µm in (E).

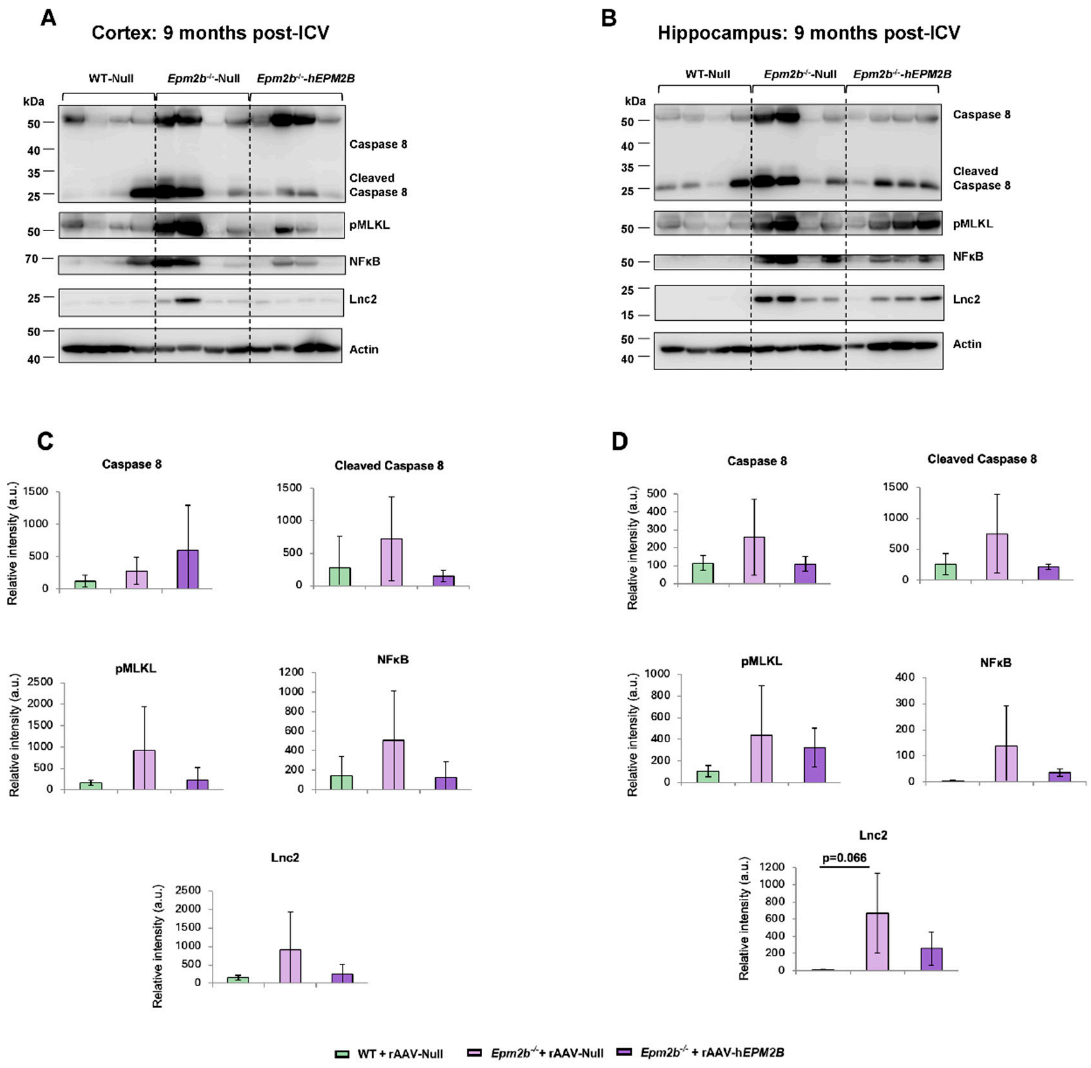

Supplemental Figure S4. Levels of neuroinflammatory markers in the cortex and hippocampus 9 months post-ICV injection of rAAV-Null or rAAV-h*EPM2B* in WT and *Epm2b*$^{-/-}$ mice Protein levels of caspase-8 (full length and cleaved), phospho (Ser345)-MLKL (pMLKL), NFκB, lipocalin 2 (Lnc2) and actin were assessed by Western blot of cortical (panel A) and hippocampal extracts (panel B) from WT mice injected with rAAV-Null and *Epm2b*$^{-/-}$ mice treated with either rAAV-h*EPM2B* or rAAV-Null, 9 months post-ICV injection. Molecular weight markers are shown on the left. Densitometric quantification of the blots was performed as described in the Materials and Methods section, with values normalized to actin and represented as arbitrary units (a.u.) for the cortex (panel C) and hippocampus (panel D). Four independent

samples from each genotype were analyzed. Results are expressed as means +/− standard deviation (SD). Differences between paired samples were analyzed by two-tailed Student's *t*-tests using GraphPad Prism version 5.0 statistical software (La Jolla, CA, USA).
